# Distinct tau filament folds in human *MAPT* mutants P301L and P301T

**DOI:** 10.1038/s41594-025-01575-9

**Published:** 2025-05-29

**Authors:** Manuel Schweighauser, Yang Shi, Alexey G. Murzin, Holly J. Garringer, Ruben Vidal, Jill R. Murrell, M. Elena Erro, Harro Seelaar, Isidro Ferrer, John C. van Swieten, Bernardino Ghetti, Sjors H. W. Scheres, Michel Goedert

**Affiliations:** 1 Medical Research Council (MRC) Laboratory of Molecular Biology, Cambridge, UK; 2 Department of Pathology of the First Affiliated Hospital and School of Brain Science, Zhejiang University, Hangzhou, China; 3 Department of Pathology and Laboratory Medicine, Indiana University School of Medicine, Indianapolis, IN USA; 4 Department of Pathology and Laboratory Medicine, Children’s Hospital of the University of Pennsylvania, Philadelphia, PA USA; 5 Department of Neurology, Hospital Universitario de Navarra, Brain Bank Navarra Biomed, Pamplona, Spain; 6 Department of Neurology, Erasmus University, Rotterdam, the Netherlands; 7 Department of Pathology and Experimental Therapeutics, University of Barcelona, Barcelona, Spain

**Keywords:** Cryoelectron microscopy, Diseases

## Abstract

Mutations in *MAPT*, the tau gene, give rise to frontotemporal dementia and parkinsonism linked to chromosome 17 (FTDP-17), with abundant filamentous tau inclusions in brain cells. Mutations that encode missense variants of residue P301 are the most common and result in the formation of filamentous inclusions made of mutant four-repeat tau. Here we report the cryo-electron microscopy structures of tau filaments from five individuals belonging to three different families with mutation P301L and from one individual from a family with mutation P301T. A distinct three-lobed tau fold resembling the two-layered fold of Pick’s disease was present in the individuals with P301L tau. Two different tau folds were found in the individual with mutation P301T, the less abundant of which was a variant of the three-lobed fold. The major P301T tau fold was V-shaped, with partial similarity to the four-layered tau folds of corticobasal degeneration and argyrophilic grain disease.

## Abstract (web summary)

Schweighauser, Shi, Murzin and colleagues report cryo-EM structures of tau filaments from individuals with P301L or P301T MAPT mutations. P301L tau filaments adopted a distinct three-lobed fold, while P301T filaments had either a variant of the three-lobed fold or a V-shaped fold.

## Main

In the adult human brain, six tau isoforms are expressed from a single *MAPT* gene through alternative mRNA splicing^1^. They differ by the inclusion or exclusion of two exons near the N terminus (exons 2 and 3) and a single exon near the C terminus (exon 10) of the protein. Exon 10 encodes a repeat of 31 amino acids, and its inclusion gives rise to three isoforms with four repeats (4R). The other three isoforms lack exon 10 expression and have three repeats (3R). These repeats and adjoining sequences constitute the microtubule-binding domains of tau. Part of this sequence also forms the core of assembled tau in neurodegenerative diseases, indicating that the physiological function of microtubule binding and the pathological assembly into amyloid filaments are mutually exclusive.

Mutations in *MAPT* lead to the formation of filamentous inclusions that are made of either 3R, 4R or 3R + 4R tau^2^. Mutations that cause the relative overproduction of wild-type 3R or 4R tau result in the deposition of 3R tau with the Pick fold^3^ or 4R tau with the argyrophilic grain disease fold^4^. Filamentous inclusions of 3R + 4R tau associated with missense mutations V337M and R406W adopt the Alzheimer fold^5^.

Missense mutations of residue P301 (P301L, P301S and P301T)^6–9^ are the most common mutations associated with FTDP-17. Most individuals with mutations P301L and P301S present with behavioral-variant frontotemporal dementia (FTD), with or without parkinsonism^10–13^. Mutation P301T has been reported to cause a clinicopathological picture of type III globular glial tauopathy (GGT)^9,14^. Mutation P301L has also been shown to give rise to GGT. All three mutations cause 4R tauopathies with abundant filamentous tau inclusions in nerve cells and glial cells. Only mutant tau is deposited in individuals with the P301L mutation^15,16^. In vitro experiments have shown that, of the *MAPT* mutations tested, P301L and P301S tau had the least potential to promote microtubule assembly and the greatest ability to form heparin-induced filaments^8,17–21^. It has been suggested that mutations of residue P301 destabilize local structure and expose the sequence ^306^VQIVYK^311^ (ref. ^22^), which is necessary for the assembly of tau into filaments^23^. Seeds of assembled recombinant P301L tau nucleate P301L, but not wild-type, tau^24^. P301L and P301S are also the most widely used mutations in transgenic mouse models of tauopathies, because their overexpression gives rise to robust phenotypes consisting of tau hyperphosphorylation, filament formation and neurodegeneration^25–28^. In these models, the assembly into tau filaments is necessary for neurodegeneration, because overexpression of human P301S tau without ^306^VQIVYK^311^ does not result in neurodegeneration^29^.

Heterozygous point mutations that give rise to an amino acid substitution at position 301 result in approximately 75% of total tau protein being wild type and 25% being mutant. In addition, recombinant P301L and P301S tau proteins have been shown to have a partial loss of microtubule polymerization function^8,17^. Loss of function of tau has been proposed to be the cause of FTDP-17 (ref. ^30^). Alternatively, the ordered assembly into filaments made of mutant tau is the gain-of-toxic function mechanism that gives rise to FTDP-17 (ref. ^31^).

By cryo-electron microscopy (cryo-EM), we reported different structures of tau filaments extracted from the brains of transgenic mice overexpressing human P301S tau downstream of the *Thy1* or the prion protein promoter^32^. The cryo-EM structure of P301L tau filaments extracted from the brains of rTg4510 transgenic mice has also been reported^33^. Here, we present the cryo-EM structures of tau filaments extracted from the cerebral cortex of individuals with the missense mutations P301L tau and P301T tau. Distinct tau folds were observed for both mutations, suggesting that mutations of residue P301 in tau lead to diseases that differ from sporadic tauopathies.

## Results

### Structures of filaments from five individuals with the *MAPT* mutation encoding P301L tau

**Fig. 1:**
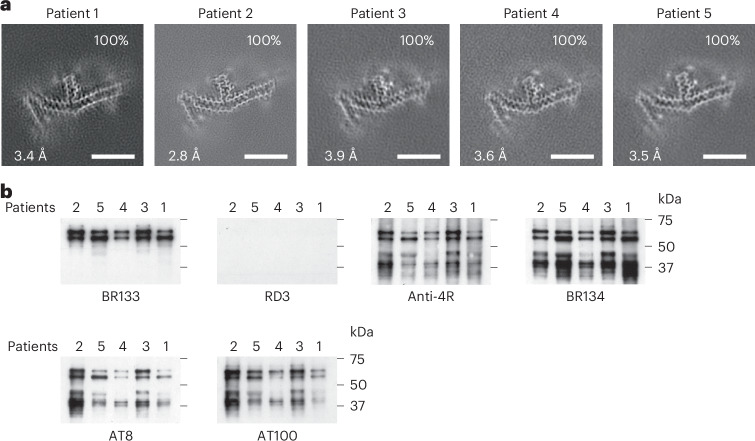
*MAPT* mutation encoding P301L tau: cryo-EM cross-sections of tau filaments and immunoblotting. **a**, Cross-sections through the cryo-EM reconstructions, perpendicular to the helical axis and with a projected thickness of approximately one rung, are shown for the parietal cortex from patients 1 and 2 and the temporal cortex from patients 3–5. Resolutions (in Å) and percentages of filament types are indicated at the bottom left and top right, respectively. All filaments had the three-lobed fold of assembled P301L tau. Scale bars, 10 nm. **b**, Immunoblotting of sarkosyl-insoluble tau from the parietal cortex of patients 1 and 2 and the temporal cortex of patients 3–5. Phosphorylation-independent anti-tau antibodies BR133, RD3, anti-4R and BR134 as well as phosphorylation-dependent anti-tau antibodies AT8 and AT100 were used. Two major tau bands of 64 and 68 kDa were labeled by all antibodies, except RD3, indicating the presence of hyperphosphorylated 4R, but not 3R, tau. Source data

**Fig. 2:**
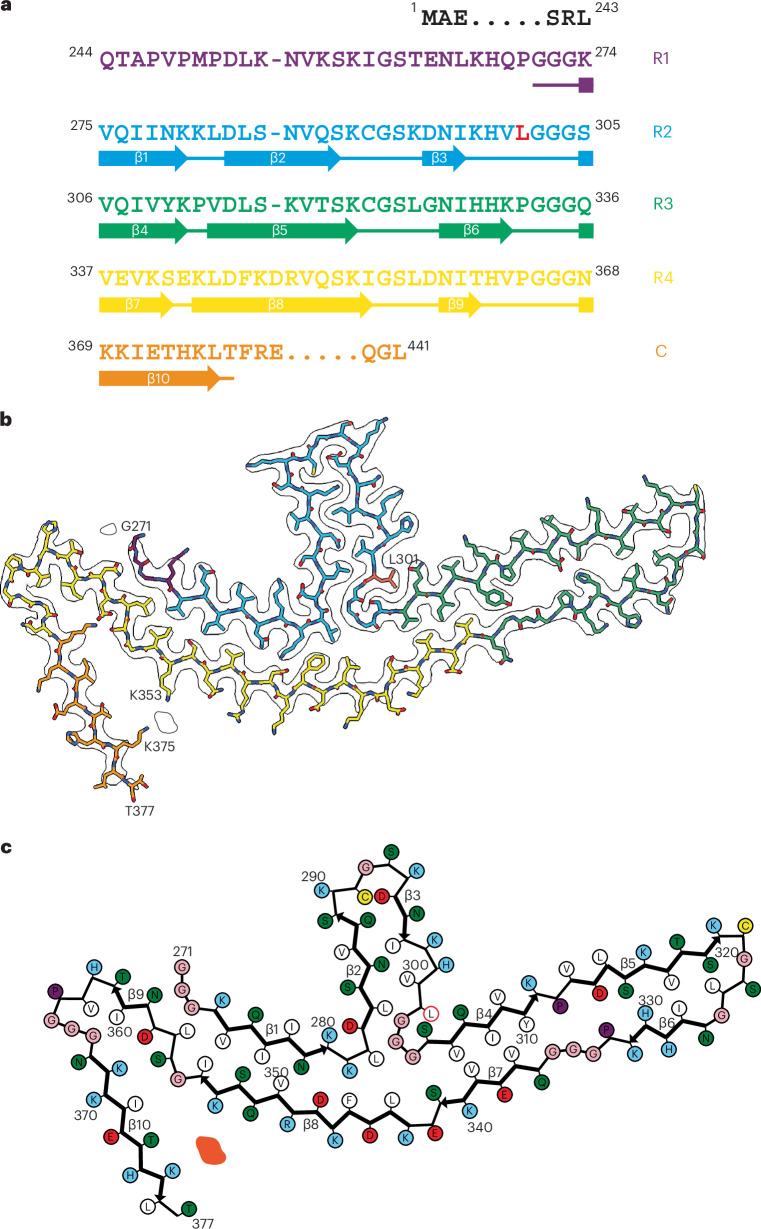
*MAPT* mutation encoding P301L tau: cryo-EM structure of tau filaments. **a**, Sequence of repeats R1–R4 of tau (residues 244–368). The core structure of tau filaments extends from G271 to T377 and comprises ten β-strands (β1–β10, shown as thick arrows; loops are shown as thin lines). Residue L301 is highlighted in red. **b**, Sharpened cryo-EM map of tau filaments from the parietal cortex of patient 2, with the atomic model overlaid. Residues in R1–R4 and the sequence after R4 are colored purple, blue, green, gold and orange, respectively. Residue L301 is labeled, together with the N-terminal G271 and the C-terminal T377 of the ordered core, as well as K353 and K375, which coordinate a nonproteinaceous density. **c**, Schematic of the P301L tau filament fold. Negatively charged residues are shown in red, positively charged residues are in blue, polar residues are in green, nonpolar residues are in white, sulfur-containing residues are in yellow, prolines are in purple, and glycines are in pink. Thick arrows indicate β-strands (β1–β10). An internal additional density is shown in orange. Residue L301 is circled in red.

**Extended Data Fig. 1:**
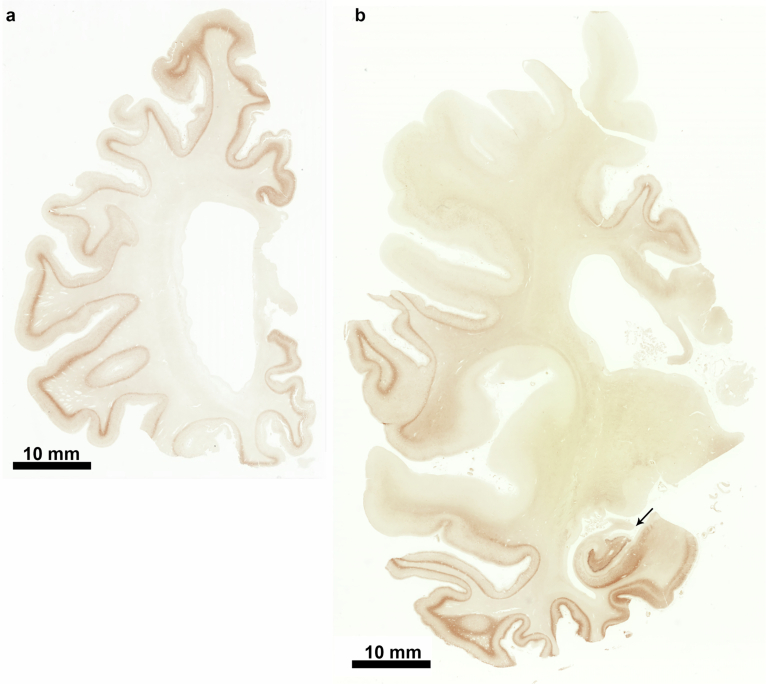
*MAPT* mutation encoding P301L tau: Tau inclusions in coronal brain sections from case 1. **a**, Coronal section of frontal lobe stained with phosphorylation-dependent anti-tau antibody AT8. Note that all gyri are strongly stained. Scale bar, 10 mm. **b**, Coronal section of parietal and temporal lobes stained with phosphorylation-dependent anti-tau antibody AT8. Note that not all gyri are labelled in the parietal lobe (upper part of the section). In the temporal lobe, inferior gyri and the hippocampus (arrowed) are strongly stained (lower part of the section). Scale bar, 10 mm.

**Extended Data Fig. 2:**
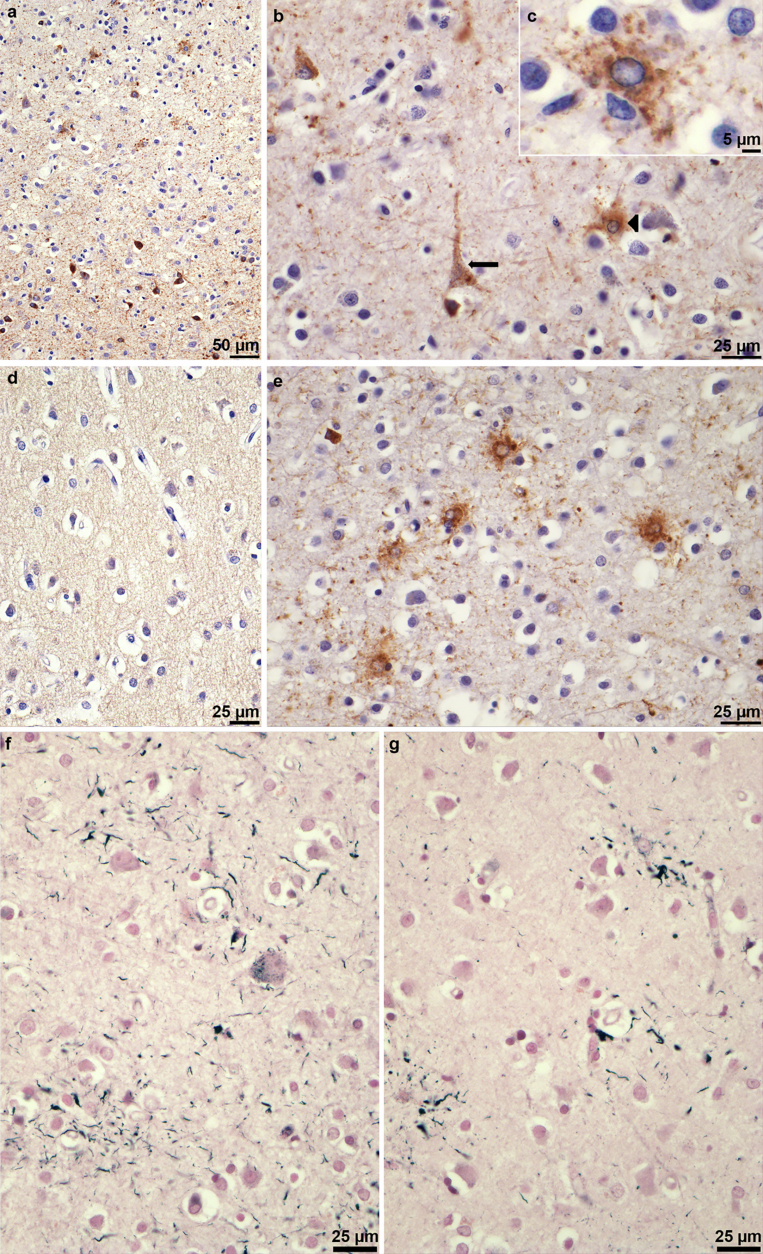
*MAPT* mutation encoding P301L tau: Tau inclusions in parietal cortex (postcentral gyrus) from case 1. The following anti-tau antibodies were used: AT8 (**a**,**b**,**c**); RD3 (**d**); RD4 (**e**). Gallyas-Braak silver staining was used in (**f**,**g**). **a**, AT8-positive nerve cells (lower part of the image) and astrocytes (upper part of the image). Scale bar, 50 μm. **b**, AT8-positive nerve cell (arrow) and AT8-positive astrocyte (arrowhead). Cell bodies and processes are labelled. Scale bar, 25 μm. **c**, An astrocyte with AT8-positive cytoplasm and processes and AT8-negative nucleus. Scale bar, 5 μm. **d**, No labelling of neurons or astrocytes by RD3. Scale bar, 25 μm. **e**, Astrocytes are labelled by RD4 (this case had more tau-immunoreactive astrocytes than neurons). Scale bar, 25 μm. **f**, Nerve cells and their processes are Gallyas-Braak silver-positive. Scale bar, 25 μm. **g**, Astrocytic processes are Gallyas-Braak silver-positive. Scale bar, 25 μm.

**Extended Data Fig. 3:**
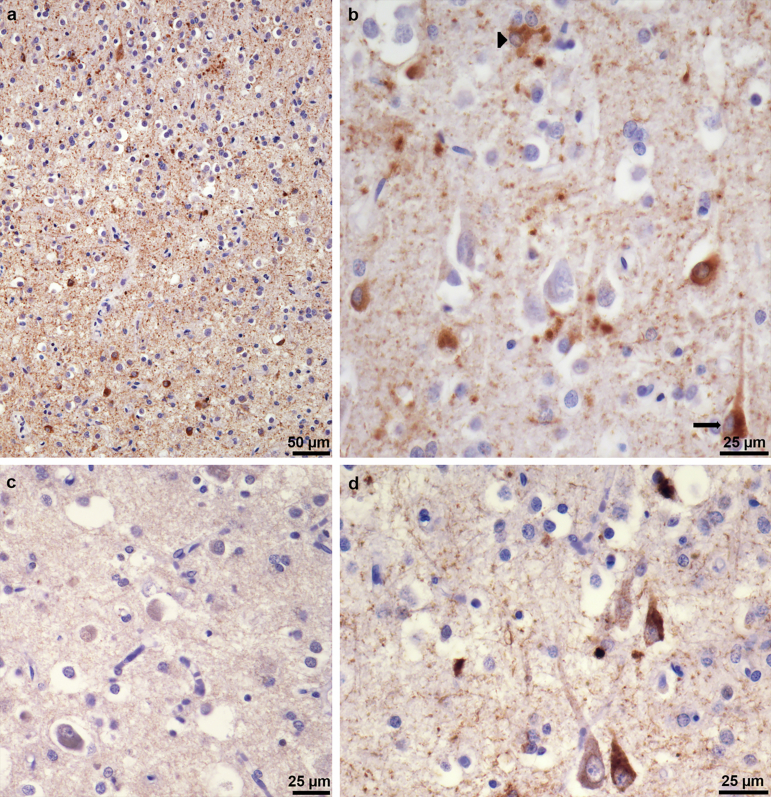
*MAPT* mutation encoding P301L tau: Tau inclusions in parietal cortex (postcentral gyrus) from case 2. The following anti-tau antibodies were used: AT8 (**a**,**b**); RD3 (**c**); RD4 (**d**). **a**, AT8-positive nerve cells (lower part of the image) and astrocytes (upper part of the image). Scale bar, 50 μm. **b**, AT8-positive nerve cell (arrow) and AT8-positive astrocyte (arrowhead). Some neuropil elements are also AT8-immunoreactive. Scale bar, 25 μm. **c**, No labelling of neurons or astrocytes by RD3. Scale bar, 25 μm. **d**, Nerve cells and neuropil threads are labelled by RD4 (this case had more tau-immunoreactive neurons than astrocytes). Scale bar, 25 μm.

**Extended Data Fig. 4:**
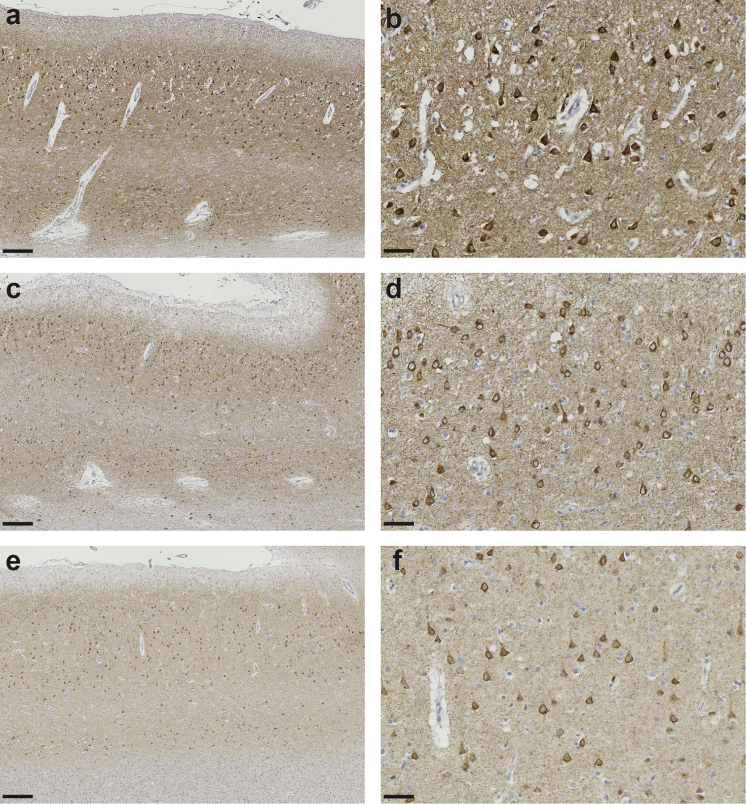
*MAPT* mutation encoding P301L tau: Tau inclusions in temporal cortex from cases 3–5. (**a-f**), Staining with phosphorylation-dependent anti-tau antibody AT8, showing numerous positive neuronal and glial cell inclusions in P301L tau cases 3 (**a**,**b**), 4 (**c**,**d**) and 5 (**e**,**f**). Scale bars: 200 μm (**a**,**c**,**e**); 50 μm (**b**,**d**,**f**).

We determined the structures of tau filaments from the cerebral cortex of five individuals belonging to three different families with the *MAPT* mutation encoding P301L tau (Figs. 1 and 2). We have no data to suggest that these families were related, but this remains to be shown unambiguously. Parietal cortex was used for patients 1 and 2, and temporal cortex was used for patients 3–5. All individuals had only tau filaments made of an identical single protofilament, with resolutions of 2.8–3.9 Å (Fig. 1a). For the sharpened map in Fig. 2, we used tau filaments that were extracted from the parietal cortex of patient 2, as this gave the highest resolution. All five individuals had a C-to-T nucleotide substitution in the second position of codon 301 (CCG to CTG, on one allele), resulting in a P301L change. The clinical diagnosis of all patients was behavioral-variant FTD. By immunoblotting of sarkosyl-insoluble fractions with BR133, RD3, anti-4R, BR134, AT8 and AT100, strong tau bands of 64 and 68 kDa were observed with all antibodies, except RD3, indicating the presence of hyperphosphorylated 4R, but not 3R, tau (Fig. 1b). Assembled tau was truncated at the N terminus, as judged by the presence of additional lower-molecular-weight tau bands with anti-4R, BR134, AT8 and AT100 but not BR133. Tau filaments form from full-length protein and consist of a core and a fuzzy coat^2^. Because the latter is unstructured, it is more prone to proteolysis. This could affect filament stability. By immunohistochemistry, abundant inclusions of hyperphosphorylated 4R tau were present in nerve cells and glial cells, chiefly astrocytes (Extended Data Figs. 1–4). They were Gallyas–Braak silver positive. Globular glial tau inclusions were not observed.

Cryo-EM showed that the tau protofilament spans residues G271–T377 and adopts a double-layered, three-lobed fold (Fig. 2). The cryo-EM density at residue 301 is consistent with a leucine side chain. Unlike the known structures of wild-type 4R tau filaments, which are folded into either three- or four-layered structures^4,34^, the three-lobed fold of P301L tau consists of a stem of two-layered cross-β structure and two hairpin-like arms that are connected at a common junction. The short arm is made of residues 282–303, whereas the long arm comprises residues 304–342. The stem is composed of two segments made of residues 271–281 and 343–362, which are packed against each other. The C-terminal segment, which consists of residues 363–377 of tau, folds back onto the stem, from which it is separated by a nonproteinaceous density. The latter probably corresponds to a negatively charged cofactor of unknown identity, which is coordinated by the positively charged side chains of K353 and K375. It is reminiscent of the nonproteinaceous densities in the ex vivo structures of other human brain tau filaments^2^. At the three-lobed junction, there is a solvent-filled cavity surrounded by tau residues L282, G304, S341 and L344.

**Extended Data Fig. 5:**
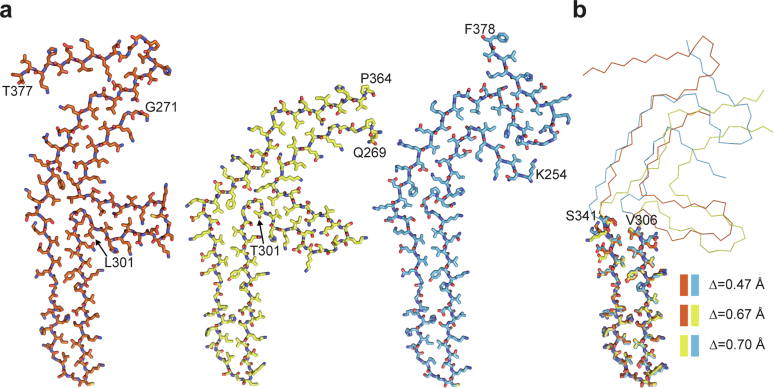
Comparison of the three-lobed folds of P301L and P301T tau filaments with the Pick tau fold. **a**, Side-by-side comparison of the three-lobed folds of P301L (left, orange) and type II P301T (middle, yellow) tau filaments with the Pick tau fold (right, blue) (PDB:8P34). **b**, Overlay of the long arm substructures (residues V306-S341) shown as sticks. Backbone root mean square deviation values Δ were calculated for each pair of substructures (colour coded as in panel **a**). The remainder of the three folds is shown as backbone.

Except for the short arm, the three-lobed fold of P301L tau resembles the Pick fold made of 3R tau^3,35^, with the conformation of the long hairpin-like arm (residues 306–341) being nearly identical to that of the same region in the Pick tau fold (Extended Data Fig. 5). The similarity of the three-lobed fold to the Pick fold also extends to the stem region, where residues 347–358 adopt similar backbone conformations and form analogous interfaces with the opposite segments, which are made of residues 275–281 of the former and residues 261–267 of the latter. Residues N279 and K281 in the P301L tau fold occupy equivalent positions to residues N265 and K267 in the Pick fold (Extended Data Fig. 5a,b).

As no structure resembling that of the short arm is present in the known filament structures of wild-type tau, its formation is probably the result of the P301L mutation stabilizing the local conformation at the R2–R3 junction. Mutation of this proline results in the formation of an additional hydrogen bond between the main chains of adjacent tau molecules. The side chain of L301 is buried between the side chains of S305 and Q307 on the long arm, the polar groups of which are engaged in hydrogen bonding interactions (Fig. 2b). The local structure at L301 is complementary to the structure of the first intermediate amyloid in the assembly of recombinant tau fragment (297–391) into either the Alzheimer or the chronic traumatic encephalopathy tau fold^36^ and can be formed on the back of it.

### Structures of filaments from an individual with a *MAPT* mutation encoding P301T tau

**Fig. 3:**
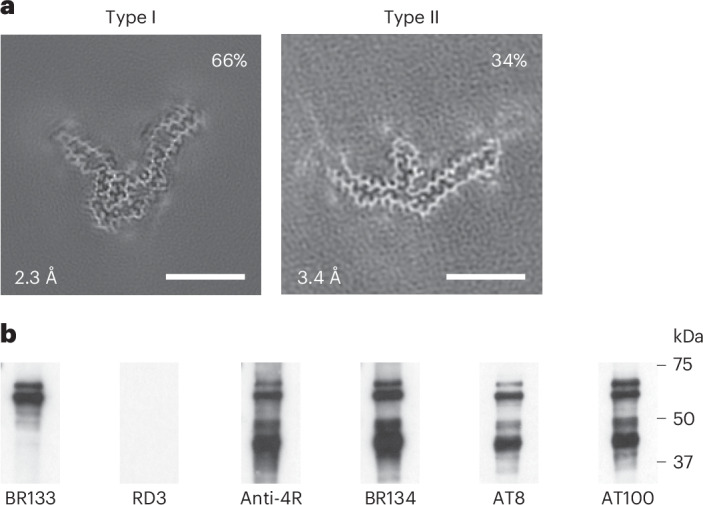
*MAPT* mutation encoding P301T tau: cryo-EM cross-sections of tau filaments and immunoblotting. **a**, Cross-sections through the cryo-EM reconstructions, perpendicular to the helical axis and with a projected thickness of approximately one rung, are shown for the frontal cortex. Resolutions (in Å) and percentages of filament types are indicated at the bottom left and top right, respectively. Type I tau filaments comprised 66% of tau filaments, and type II comprised 34% of tau filaments. Scale bars, 10 nm. **b**, Immunoblotting of sarkosyl-insoluble tau from the frontal cortex. Phosphorylation-independent anti-tau antibodies BR133, RD3, anti-4R and BR134 as well as phosphorylation-dependent antibodies AT8 and AT100 were used. Two major tau bands of 64 and 68 kDa were labeled by all antibodies, except RD3, indicating the presence of hyperphosphorylated 4R, but not 3R, tau. Source data

**Fig. 4:**
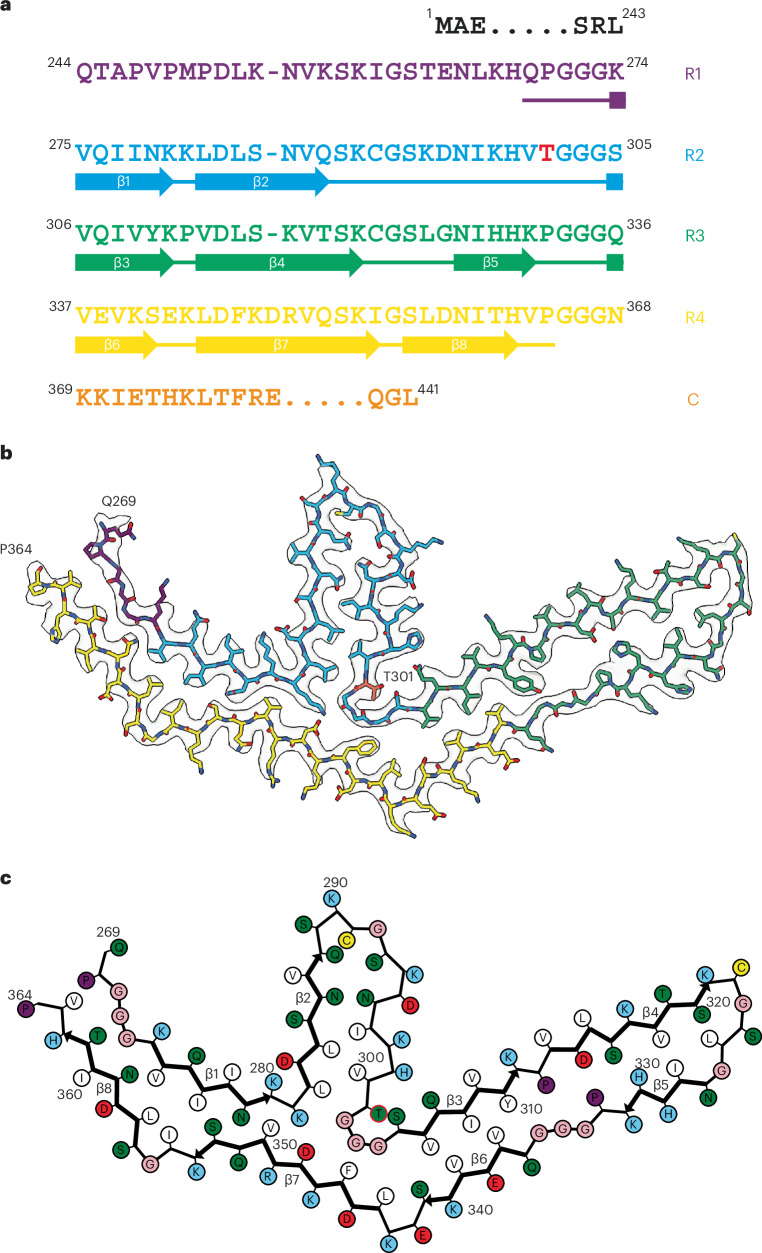
*MAPT* mutation encoding P301T tau: cryo-EM structure of type II tau filaments. **a**, Sequence of repeats R1–R4 of tau (residues 244–368). The core structure of tau filaments extends from N269 to P364 and comprises eight β-strands (β1–β8, shown as thick arrows; loops are shown as thin lines). Residue T301 is highlighted in red. **b**, Sharpened cryo-EM map of type II tau filaments from the frontal cortex, with the atomic model overlaid. Residues in R1–R4 and the sequence after R4 are colored purple, blue, green, gold and orange, respectively. Residue T301 is labeled and so are the N-terminal residue Q269 and the C-terminal residue P364 of the ordered core. **c**, Schematic of the type II P301T tau filament fold. Negatively charged residues are shown in red, positively charged residues are in blue, polar residues are in green, nonpolar residues are in white, sulfur-containing residues are in yellow, prolines are in purple, and glycines are in pink. Thick arrows indicate β-strands 1–8. Residue T301 is circled in red.

**Fig. 5:**
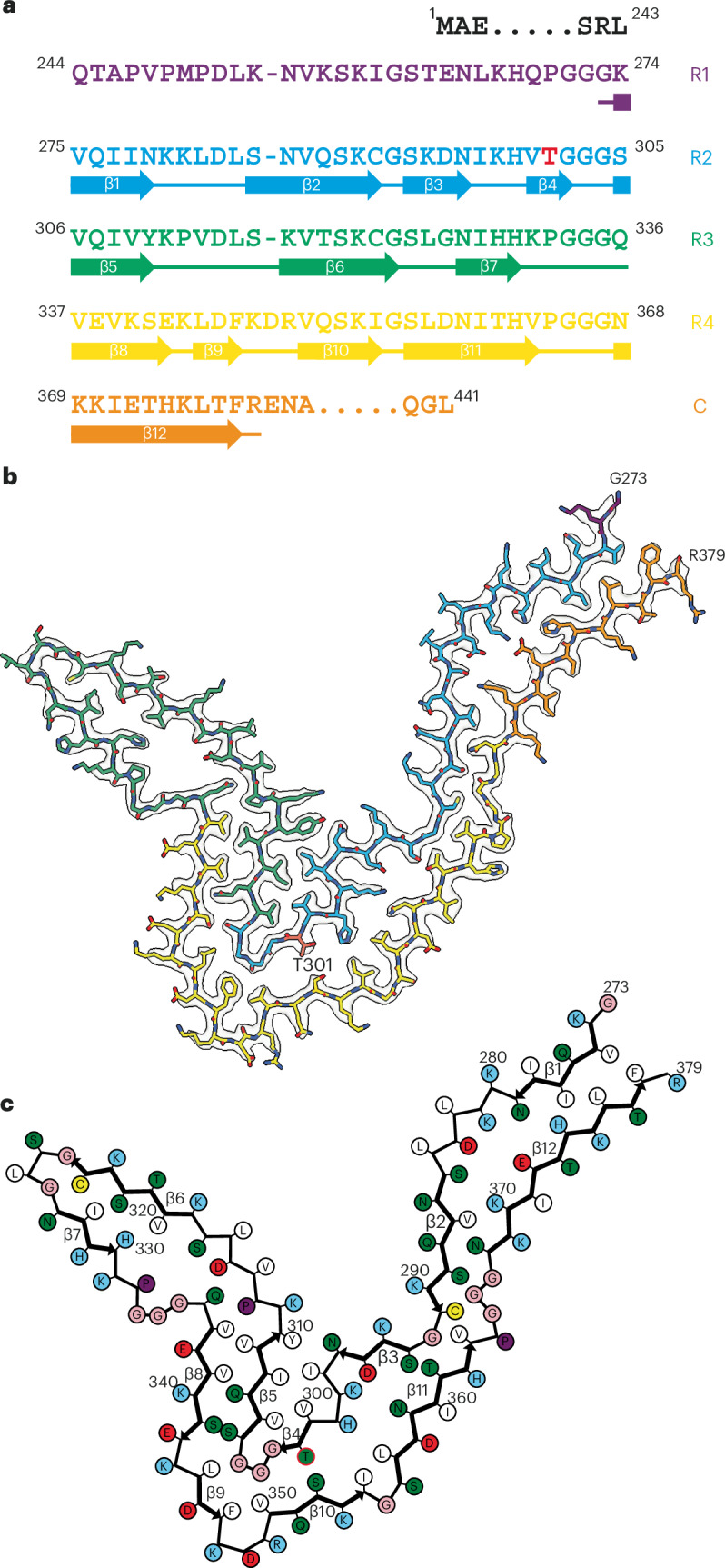
*MAPT* mutation encoding P301T tau: cryo-EM structure of type I tau filaments. **a**, Sequence of repeats R1–R4 of tau (residues 244–368). The core structure of type I tau filaments extends from G273 to R379 and comprises 12 β-strands (β1–β12, shown as thick arrows; loops are shown as thin lines). Residue T301 is highlighted in red. **b**, Sharpened cryo-EM map of type I tau filaments from the frontal cortex, with the atomic model overlaid. Residues in R1–R4 and the sequence after R4 are colored purple, blue, green, gold and orange, respectively. **c**, Schematic of the type I P301T tau filament fold. Negatively charged residues are shown in red, positively charged residues are in blue, polar residues are in green, nonpolar residues are in white, sulfur-containing residues are in yellow, prolines are in purple, and glycines are in pink. Thick arrows indicate β-strands 1–12. Residue T301 is circled in red.

We also determined the structures of tau filaments from the frontal cortex of a previously described individual^9,14^ belonging to a Spanish family with mutation P301T encoded by *MAPT* (Figs. 3–5). The clinicopathological diagnosis was GGT type III. Two types of tau filament, each made of a single protofilament, were present (Fig. 3a). By immunoblotting of the sarkosyl-insoluble fraction with BR133, RD3, anti-4R, BR134, AT8 and AT100, strong tau bands of 64 and 68 kDa were observed with all antibodies except RD3, consistent with the presence of hyperphosphorylated 4R, but not 3R, tau (Fig. 3b). Assembled tau was truncated at the N terminus, as judged by the presence of additional lower-molecular-weight tau bands with anti-4R, BR134, AT8 and AT100 but not BR133. By immunohistochemistry, abundant neuronal and glial 4R tau inclusions were present, with numerous globular glial tau inclusions in astrocytes and oligodendrocytes^14^.

By cryo-EM, type I filaments comprised approximately 66% and type II filaments around 34% of tau filaments (Fig. 3a). Even though both types of filaments are made of single protofilaments, their ordered cores have different folds. In type II P301T tau filaments, the core spans residues N269–P364 and adopts a three-lobed fold resembling that of P301L tau filaments (Fig. 4 and Extended Data Fig. 5a). This fold also consists of a stem and two hairpin-like arms, but it is shorter than the P301L fold at the C terminus. Its long arm (residues 304–342) is practically identical to that of P301L tau filaments (Extended Data Fig. 5b), but the short arm and the stem differ by their relative orientations and the packing of interior residues. In the stem, the N-terminal segment (residues 271–281) is shifted by two residues relative to the C-terminal segment (residues 343–364), forming an interface resembling that between these segments in the P301S tau filament fold of PS19 transgenic mice^32^. In the short arm, the opposite sides of the hairpin-like structure of type II P301T filaments have conformations resembling those of P301L tau filaments, but they are shifted relative to each other by two residues. These differences correlate with a local conformational difference around the mutation site at residue 301, the cryo-EM density of which is consistent with that of threonine. The side chain of T301 fits inside the turn formed by the glycine triplet (residues 302–304) and hydrogen bonds with S305 (Fig. 4b). Compared to L301, T301 is located on the other side of the side chain of S305.

In type I P301T filaments, the core spans residues G273–R379 of tau and adopts a new fold in the shape of the letter V (Fig. 5). It has a four-layered body and two-layered wings. The N-terminal half of the core sequence (residues 273–324) forms the inner side of the V-shaped fold with a sharp bend at the R2–R3 junction, whereas the C-terminal half (residues 325–379) curves around this bend on the outer side.

**Extended Data Fig. 6:**
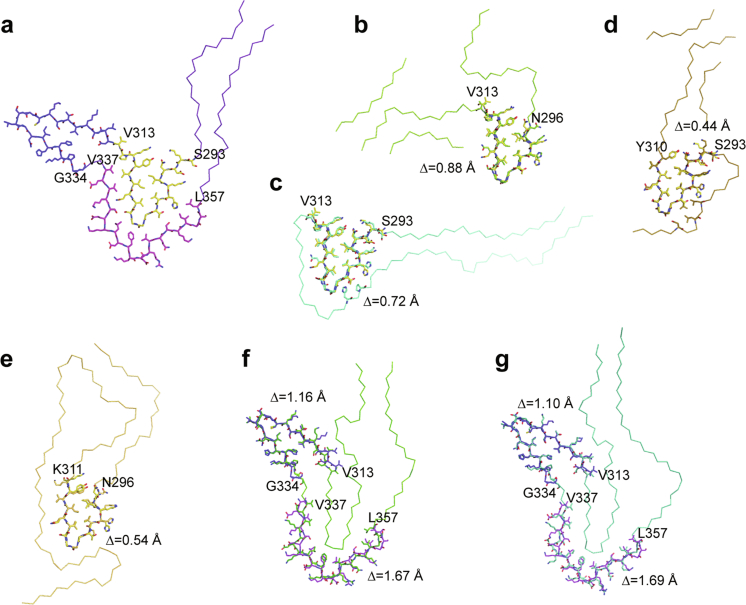
Comparison of the type I P301T tau fold with structures of tau filaments from human brains and transgenic mouse brains. **a**, Ribbon plot of the type I P301T fold. Shown as sticks and highlighted by different colours are substructures shared with other filaments. **b-g**, Overlays of the corresponding substructures are shown as sticks. Backbone root mean square deviation values Δ are shown for each overlay. **b**, P301S tau filament fold from mouse line Tg2541 (PDB:8Q96). **c**, P301S tau filament fold from mouse line PS19 (PDB:8Q92). **d**, P301L tau filament fold from mouse line rTg4510 (PDB:8WCP). **e**, Globular glial tauopathy-progressive supranuclear palsy tau (GPT) fold (PDB:7P6A). **f**, Corticobasal degeneration (CBD) fold (PDB:6TJO). **g**, Argyrophilic grain disease (AGD) fold (PDB:7P6D).

The conformation of the R2–R3 junction in type I P301T filaments is different from that of type II P301T filaments, but it is almost identical to the conformations of the R2–R3 junction in filaments from transgenic mouse lines expressing human tau with mutations P301L and P301S (refs. ^32,33^) (Extended Data Fig. 6). A similar conformation of this junction was also observed in the globular glial tauopathy-progressive supranuclear palsy tau fold of wild-type 4R tau^4^. Missense mutations in P301 stabilize this conformation by creating an additional hydrogen bond to the main chain of an adjacent molecule. The side chain of T301 is on the outside surface of this common substructure and makes a solvent-mediated interaction with residue S352 of R4.

The type I P301T tau fold also displays local similarities with the corticobasal degeneration and argyrophilic grain disease tau folds by sharing with them the substructures 312–333 in R3 and 337–357 in R4. In all three folds, residues 312–333 form a compact semi-detached unit, whereas residues 337–357 wrap around the R2–R3 junction. However, in the type I P301T tau filaments, the substructure from R4 makes interactions with the R2–R3 junction that have an inside–out conformation compared to the corticobasal degeneration and argyrophilic grain disease folds. In addition, in the type I P301T and in the corticobasal degeneration folds, there are similar interfaces between the N-terminal (residues 275–281) and C-terminal (residues 372–378) segments, which are made of the same residues but are shifted relative to each other.

## Discussion

FTDP-17 is a heterogeneous clinicopathological entity, with the long arm of chromosome 17 harboring both the progranulin gene and *MAPT*. Heterozygous loss-of-function mutations in the progranulin gene give rise to FTDP-17 with abundant TAR DNA-binding protein 43 (TDP-43) inclusions^37–40^, whereas heterozygous gain-of-toxic function mutations in *MAPT* give rise to FTDP-17 with abundant tau inclusions^6,7,41^. These different molecular constituents of the filamentous inclusions suggest that progranulin gene and *MAPT* mutations lead to different diseases. We have previously shown that cases of FTDP-17 caused by *MAPT* mutations can be subdivided and that the different filament folds of mutant tau are identical to those of sporadic tauopathies^3–5^. The structures presented here, which are different from those of sporadic tauopathies, provide additional insights into this heterogeneity.

The atomic structures of tau filaments from the brains of five individuals with a P301L tau mutation belonging to three different families and the structures of tau filaments from the brain of an individual with a P301T mutation are distinct from any of the structures described previously for individuals with *MAPT* mutations, including V337M, R406W, ΔK281 and intron 10 mutations +3 and +16 (refs. ^3–5^). Whereas mutations V337M and R406W lead to the same tau filaments as in Alzheimer’s disease^5^, ΔK281 gives rise to tau filaments with the Pick fold^3^, and intron 10 mutations +3 and +16 lead to filaments with the argyrophilic grain disease fold^4^. Mutations P301L and P301T in tau give rise to folds that are distinct from those described previously for sporadic and familial tauopathies.

It has been proposed to retire the term FTDP-17, based on the assumption that frontotemporal lobar degeneration (FTLD)-tau with *MAPT* mutations are the familial forms of sporadic FTLD-tau^13^. However, the findings reported here do not support this proposal. We suggest instead to refer to patients with *MAPT* mutations as having FTDP-17T to distinguish them from patients with FTDP-17 with progranulin gene mutations.

Distinct tau folds define different diseases, but the same fold can be found in multiple diseases, for instance, chronic traumatic encephalopathy, subacute sclerosing panencephalitis, amyotrophic lateral sclerosis–parkinsonism–dementia complex and vacuolar tauopathy^2,42^. If this is also true of FTDP-17T, the mutations P301L tau and P301T tau do belong to separate familial tauopathies. Moreover, tau filaments from an individual with mutation P301T tau, which gives rise to GGT type III^14^, have folds that are distinct from those of sporadic GGT types I and II^4^. We previously reported that the tau filaments from a patient with sporadic GGT type III are straight, precluding the determination of their structures^4^. GGT type III may thus be a different disease than types I and II, and filament structures may differ between sporadic and inherited forms of disease. Individuals with P301L tau and a clinicopathological diagnosis of GGT have been described^11–13^, but their tau filament structures remain to be determined.

Like the tau filaments from progressive supranuclear palsy, all the tau filaments from individuals with P301L and P301T tau were made of single protofilaments. While we observed the same tau fold in filaments extracted from the cerebral cortex of five individuals with the mutation P301L tau, we had only access to brain material from a single individual with the mutation P301T tau, for whom we observed two different protofilament folds. Intriguingly, the type II P301T fold resembled the P301L tau fold. It will be interesting to see whether additional individuals with the *MAPT* mutation encoding P301T tau have the same folds as those shown here.

Surprisingly, the three-lobed folds of 4R tau with the P301L or P301T folds bear similarity to the Pick fold of 3R tau^35^. In human individuals with the P301L tau mutation, characteristic mini Pick-like bodies have been described by light microscopy, especially in the dentate gyrus^11,43,44^. However, unlike Pick bodies^45^, the 4R tau inclusions from an individual with mutation P301L tau were Gallyas–Braak silver positive.

Filaments of tau from the brains of individuals with a P301 mutation are made only of mutant tau, unlike filaments from individuals with mutations V337M and R406W, which adopt the Alzheimer tau fold and can consist of a mixture of wild-type and mutant proteins^5^. Wild-type human tau may be unable to form the P301L or P301T folds, even in the presence of seeds made of either P301L or P301T tau. The observation that the P301L tau folds are different from the folds of wild-type tau may explain why the PET ligand [^18^F]-flortaucipir, which binds with high affinity to the Alzheimer tau fold, showed only little binding in individuals with the mutation P301L encoded by *MAPT*^46–49^.

Different conformations of the R2–R3 junctions in the P301L and P301T tau folds of filaments extracted from human brains, together with work on filaments extracted from the brains of mice transgenic for human P301S or P301L tau^32,33^, underscore the polymorphic nature of filaments assembled from tau with a P301 mutation. The structures of P301L tau filaments from human brains are different from those of P301L tau filaments from the transgenic mouse line rTg4510 (ref. ^34^). The latter also differ from the structures of tau filaments from mouse lines expressing human mutant P301S tau^32^.

Tau filament cores from transgenic mouse lines rTg4510, Tg2541 and PS19 are shorter than those of tau filaments extracted from human brains^2,32,33^. Unlike tau filaments from human brains, mouse brain tau filament cores do not contain the whole of R4 or the sequence after R4. Of note, the P301L tau filaments from the mouse line rTg4510 and the P301S tau filaments from line Tg2541 share a large substructure outside the mutation site that has not been observed in the tau filaments extracted from human brains. The structures of tau filaments from humans with P301S tau are not known. It remains to be determined whether transgenic mouse lines can be produced that form the same tau filament folds as those found in human brains.

Mutation of P301 in tau has led to the development of transgenic mouse models that develop hyperphosphorylation of tau, filament formation and neurodegeneration^25–29^. However, the observed polymorphisms in tau filaments with P301 mutations suggest that such models should be used with caution in the study of tau pathologies of either Alzheimer’s disease or sporadic 4R tauopathies, such as progressive supranuclear palsy. The challenge is to produce mouse lines expressing human P301L or P301T tau with the same filament structures as those found in human brains.

## Methods

### Ethics

The studies carried out at Indiana University (1011003338) (patients 1 and 2 with the *MAPT* mutation encoding P301L tau), Rotterdam University (patients 3–5 with the *MAPT* mutation encoding P301L tau) and the University of Barcelona (patient with the *MAPT* mutation encoding P301T tau) were approved through the ethical review processes at each university’s institutional review board. The cryo-EM study was approved by the Cambridgeshire Research Ethics Committee (909/HO308/163). Informed consent was obtained from the patients’ next of kin.

### Individuals with the *MAPT* mutation encoding P301L tau

We used parietal cortex from two individuals (patients 1 and 2) belonging to two separate US families with the *MAPT* mutation encoding P301L tau. Patient 1 was a female who died at the age of 62 years after a 10-year history of personality changes and cognitive impairment. Her mother died with a dementing illness, and her sister had FTD. Patient 2 was a female who died at the age of 55 years after an 11-year history of FTD. No family history of dementia was reported. We used temporal cortex from three individuals (patients 3–5) belonging to a large pedigree (family 1) from the Netherlands with the *MAPT* mutation encoding P301L tau^7,50^. A variety of symptoms developed, consistent with a diagnosis of behavioral-variant FTD. Patient 3 was a male who died at the age of 55 years after a 2-year history of FTD. His mother, brother and sister had suffered from FTD. Patient 4 was a male who died at the age of 57 years after a 4-year history of FTD. His mother and two of his brothers had FTD. Patient 5 was a male who died at the age of 64 years after an 11-year history of FTD. His mother, her brother and her sister suffered from FTD.

### Individual with the *MAPT* mutation encoding P301T tau

We used frontal cortex from a previously described male (case III.3.1. in ref. ^9^; case 1 in ref. ^14^) from a Spanish family with the *MAPT* mutation encoding P301T tau. This individual had a C-to-A nucleotide substitution in the first position of codon 301 (CCG to ACG, on one allele). At the age of 45 years, he started to develop a pyramidal syndrome with features of primary lateral sclerosis, severe supranuclear ophthalmoplegia, parkinsonism and dementia. He died at the age of 49 years with a clinical diagnosis of progressive supranuclear palsy with primary lateral sclerosis. The clinicopathological diagnosis was GGT type III. The patient’s mother died at the age of 70 years with a clinical diagnosis of probable corticobasal degeneration, and his maternal grandfather suffered from dementia and died at the age of 53 years.

### Genomic analysis

Genomic DNA was extracted from human brains with informed consent. Standard amplification reactions were done with 50 ng genomic DNA, followed by sequencing of exons 1 and 9–13 of *MAPT* with adjoining intronic sequences, as described^51^. For this study, the P301L tau mutations were confirmed in the relevant brain tissues.

### Filament extraction from the human brain

Sarkosyl-insoluble material was extracted from the parietal cortex of patients 1 and 2 with P301L tau, the temporal cortex of patients 3–5 with P301L tau and the frontal cortex of the patient with P301T tau, as described^52^. Tissues were homogenized in 20 volumes (wt/vol) of buffer A (10 mM Tris-HCl, pH 7.4, 0.8 M NaCl, 10% sucrose and 1 mM EGTA), brought to 2% sarkosyl and incubated at 37 °C for 30 min. The samples were centrifuged at 10,000*g* for 25 min, followed by centrifugation of the supernatants at 100,000*g* for 60 min. The pellets were resuspended in buffer A (700 μl per g tissue) and centrifuged at 5,000*g* for 5 min. The supernatants were diluted threefold in buffer B (50 mM Tris-HCl, pH 7.5, 0.15 M NaCl, 10% sucrose and 0.2% sarkosyl), followed by a 30-min centrifugation step at 166,000*g*. For cryo-EM, the pellets were resuspended in 150 μl per g buffer C (20 mM Tris-HCl, pH 7.4, 100 mM NaCl).

### Immunoblotting and histology

For immunoblotting, samples were resolved on 4–12% Bis-Tris gels (NuPAGE), and the primary antibodies (BR133, RD3, anti-4R, BR134, AT8 and AT100) were diluted in PBS with 0.1% Tween-20 and 5% non-fat dry milk. Molecular weight markers were purchased from Bio-Rad. Histology and immunohistochemistry were carried out as described^51^. Some sections (8 μm) were counterstained with hematoxylin–eosin. The primary antibodies were RD3, RD4 and AT8. For labeling with antibodies RD3 and RD4, tissue sections required formic acid pretreatment before heat-induced epitope retrieval. For labeling with AT8, only heat-induced epitope retrieval was used. Tissue sections were then incubated overnight at 4 °C with the primary antibodies. For labeling with monoclonal and polyclonal antibodies, the universal prediluted kit ImmPRESS HRP Universal Antibody (Horse Anti-Mouse/Rabbit IgG) Polymer Detection Kit (Vector Laboratories) and the diaminobenzidine substrate kit (Vector Laboratories) were used. Gallyas–Braak silver staining was carried out as described^53^. Detailed antibody information is provided in Supplementary Table 1.

### Electron cryo-microscopy

**Extended Data Fig. 7:**
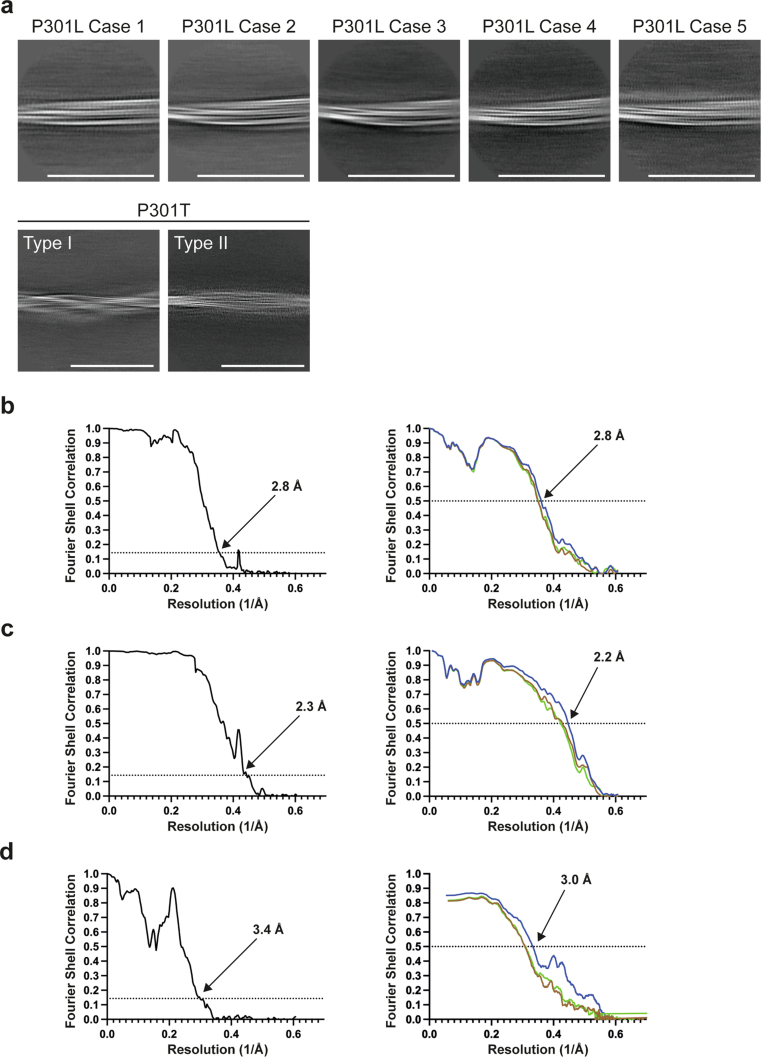
Cryo-EM 2D classification and resolution estimates. **a**, Representative 2D classification images of tau filaments from cases with *MAPT* mutations P301L and P301T. Scale bars, 50 nm. **b-d**, Solvent-corrected Fourier shell correlation (FSC) curves of cryo-EM half-maps (left panels) and model-to-map validation (right panels) for P301L tau filaments (from case 2) (**b**), P301T tau filaments type I (**c**) and P301T tau filaments type II (**d**). FSC curves for the final refined atomic model against the final cryo-EM map are shown in red; for the atomic model refined in the first half map in brown (model 1 versus half map 1); and for the refined atomic model in the first half map against the other half map in green (model 1 versus half map 2).

**Extended Data Fig. 8:**
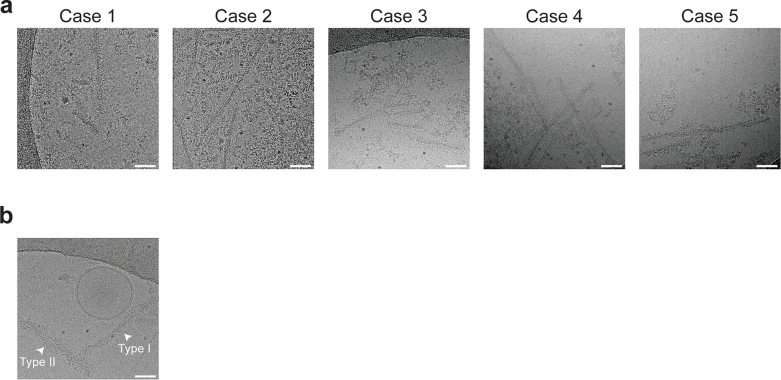
Cryo-EM images of P301L and P301T filaments. Representative motion-corrected micrographs of tau filaments from the sarkosyl-insoluble fractions of (**a**) cases 1–5 with *MAPT* mutation P301L and (**b**) the case with *MAPT* mutation P301T. Scale bar, 50 nm.

Cryo-EM grids (Quantifoil 1.2/1.3, 300 mesh) were glow discharged for 1 min using an Edwards (S150B) sputter coater. Three microliters of the sarkosyl-insoluble fractions were applied to the glow-discharged grids, followed by blotting with filter paper and plunge freezing into liquid ethane using a Vitrobot Mark IV System (Thermo Fisher Scientific) at 4 °C and 100% humidity. Cryo-EM images were acquired on a Krios G2 or G4 microscope (Thermo Fisher Scientific) operated at 300 kV. For P301L tau sample 5, movies were acquired on a Gatan K2 summit detector using a pixel size of 1.1 Å. For P301L tau samples 3 and 4, images were acquired on a Gatan K3 detector with a pixel size of 0.93 Å. For both the K2 and K3 detectors, a quantum energy filter with a slit width of 20 eV was used to remove inelastically scattered electrons. For P301L tau samples 1 and 2 and for the P301T tau sample, movies were acquired on a Falcon 4 detector using a pixel size of 0.824 Å. Images were recorded in electron event representation format^54^. See Extended Data Table 1 and Extended Data Figs. 7 and 8 for further details.

### Data processing

Datasets were processed in RELION using standard helical reconstruction^55,56^. Movie frames were gain corrected, aligned and dose weighted using RELION’s own motion correction program^57^. Contrast transfer function (CTF) was estimated using CTFFIND4.1 (ref. ^58^). Filaments were picked manually. Initial models were generated de novo from 2D class average images using relion_helix_inimodel2d^59^. Three-dimensional refinements were performed in RELION-4.0, and the helical twist and rise refined using local searches. Bayesian polishing and CTF refinement were sharpened using post-processing procedures in RELION-4.0 (ref. ^60^), and resolution estimates were calculated based on Fourier shell correlation (FSC) between two independently refined half-maps at 0.143 (ref. ^61^) (Extended Data Fig. 6). We used relion_helix_toolbox to impose helical symmetry on the post-processing maps.

### Model building and refinement

Atomic models of the P301L and P301T (type I and type II) filaments were built manually using Coot^62^. Refinements were performed using ISOLDE^63^, Servalcat^64^ and REFMAC5 (refs. ^65,66^). Models were validated with MolProbity^67^. Figures were prepared with ChimeraX^68^ and PyMOL^69^. When multiple maps of the same filament type were resolved, atomic modeling and database submission were only performed for the map with the highest resolution. The schematics in Figs. 2c and 4c were generated using the atom2svg script^70^.

### Statistics and reproducibility

No statistical method was used to predetermine sample size. No data were excluded from the analyses. The experiments were not randomized. The investigators were not blinded to allocation during experiments and outcome assessment.

### Reporting summary

Further information on research design is available in the Nature Portfolio Reporting Summary linked to this article.

## Online content

Any methods, additional references, Nature Portfolio reporting summaries, source data, extended data, supplementary information, acknowledgements, peer review information; details of author contributions and competing interests; and statements of data and code availability are available at 10.1038/s41594-025-01575-9.

## Supplementary information

Supplementary Information Supplementary Table 1.

Reporting Summary

## Source data

Source Data Fig. 1 Uncropped western blots for Fig. 1b.

Source Data Fig. 3 Uncropped western blots for Fig. 3b.

## Data Availability

Cryo-EM maps have been deposited in the Electron Microscopy Data Bank with the accession numbers EMD-51319 for P301L tau filaments from patient 2, EMD-51320 for type I P301T tau filaments and EMD-51325 for type II P301T tau filaments. The corresponding refined atomic models have been deposited in the Protein Data Bank under accession numbers 9GG0 for P301L tau filaments from patient 2, 9GG1 for type I P301T tau filaments and 9GG6 for type II P301T tau filaments. The markers in the uncropped blots were transferred from the X-ray films and annotated manually. Molecular weights were inferred from the guidelines provided by Bio-Rad. Source data are provided with this paper.

## Extended data

**Extended Data Table 1:** Cryo-EM data collection, refinement and validation statistics

	P301L	P301L	P301L	P301L	P301L	P301T	
	Case 1	Case 2	Case 3	Case 4	Case 5	Case 1	
**Data collection and processing**							
Magnification	96,000	96,000	81,000	81,000	105,000	96,000	
Voltage (kV)	300	300	300	300	300	300	
Detector	Falcon 4	Falcon 4	K3	K3	K2	Falcon 4	
Electron dose (e–/Å^2^)	30.0	30.0	32.7	33.3	42.5	42.0	
Defocus range (μm)	1.8-2.4	1.7–2.4	1.6–2.4	1.6–2.6	1.6–2.6	1.0-2.6	
Pixel size (Å)	0.824	0.824	0.93	0.93	1.1	0.824	
Initial particle images (no.)	182,321	183,040	259,362	94,149	116,182	1,073,937	
						Type I	Type II
Symmetry imposed	C1	C1	C1	C1	C1	C1	C1
Final particle images (no.)	182,321	68,559	38,300	10,457	15,649	102,697	3,795
Map resolution (Å)	3.39	2.81	3.86	3.55	3.45	2.26	3.36
FSC threshold = 0.143							
Map sharpening *B* factor (Å^2^)	−66.9	−42.2	−71.5	−57.1	−26.3	−36.0	−12.5
Helical rise (Å)	4.81	4.80	4.87	4.87	4.79	4.76	4.79
Helical twist (°)	−0.56	−0.63	−0.60	−0.62	−0.64	−1.18	−0.77
		P301L				P301T	P301T
		Case 2				Type I	Type II
**Model Refinement**							
Initial model used (PDB)		−				−	−
Model resolution (Å)		2.78				2.23	3.00
FSC threshold = 0.5							
Model composition							
Non-hydrogen atoms		2,385				3,232	2142
Protein residues		321				428	288
Ligands		0				0	0
*B* factors (Å^2^)							
Protein		56.4				41.2	79.09
R.m.s. deviations							
Bond lengths (Å)		0.0061				0.0040	0.003
Bond angles (°)		1.217				1.155	0.710
Validation							
MolProbity score		1.38				1.28	1.67
Clashscore		1.68				1.51	4.80
Poor rotamers (%)		0.00				0.00	0.00
Ramachandran plot							
Favored (%)		92.38				94.29	93.62
Allowed (%)		7.62				5.72	6.38
Disallowed (%)		0.00				0.00	0.00
PDB		9GG0				9GG1	9GG6
EMDB		51319				51320	51325
