# Supplementary material for: Distinct tau filament folds in human MAPT mutants P301L and P301T
Source: Nat Struct Mol Biol. 2025 May 29;32(8):1470–8. doi: 10.1038/s41594-025-01575-9 (PMC12350173; doi:10.1038/s41594-025-01575-9)
Supplement: Supplementary file 1 — Supplementary Table 1. [file 41594_2025_1575_MOESM1_ESM.pdf]

nature structural & molecular biology

Supplementary information

https://doi.org/10.1038/s41594-025-01575-9

Distinct tau filament folds in human *MAPT* mutants P301L and P301T

In the format provided by the authors and unedited

SUPPLEMENTARY INFORMATION

Table of contents:

- Supplementary Table 1

Supplementary Table 1. Anti-tau antibodies.

Name	Epitope	Supplier	Cat. number	Host species	Class	WB dilution	IHC dilution	Validation
BR133	N-terminus	In-house	-	Rabbit	Polyclonal	1:4,000	-	Goedert et al. 1989 Neuron 3,519-526
BR134	C-terminus	In-house	-	Rabbit	Polyclonal	1:4,000	-	Goedert et al. 1989 Neuron 3,519-526
RD3	R1/3	Millipore	05-803	Mouse	Monoclonal	1:4,000	1:3,000	Manufacturer's datasheet
RD4	R2	Millipore	05-804	Mouse	Monoclonal	-	1:100	Manufacturer's datasheet
Anti-4R	R2	Cosmo Bio	CAC-TIP-4RT-P01	Rabbit	Polyclonal	1:2,000	1:400	Manufacturer's datasheet
AT8	pS202/pT205	Thermo Fisher	MN1020	Mouse	Monoclonal	1:1,000	1:1,000	Manufacturer's datasheet
AT100	pT212/pS214	Thermo Fisher	MN1060	Mouse	Monoclonal	1:500	-	Manufacturer's datasheet

WB, Western blot; IHC, immunohistochemistry.
