# Supplementary material for: Distinct tau filament folds in human MAPT mutants P301L and P301T
Source: Nat Struct Mol Biol. 2025 May 29;32(8):1470–8. doi: 10.1038/s41594-025-01575-9 (PMC12350173; doi:10.1038/s41594-025-01575-9)
Supplement: Supplementary file 2 — Reporting Summary [file 41594_2025_1575_MOESM2_ESM.pdf]

nature portfolio

Corresponding author(s): Sjors H. W. Scheres, Michel Goedert

Last updated by author(s): Mar 18, 2025

Reporting Summary

Nature Portfolio wishes to improve the reproducibility of the work that we publish. This form provides structure for consistency and transparency in reporting. For further information on Nature Portfolio policies, see our Editorial Policies and the Editorial Policy Checklist.

Statistics

For all statistical analyses, confirm that the following items are present in the figure legend, table legend, main text, or Methods section.

n/a	Confirmed	
☐	☒	The exact sample size (*n*) for each experimental group/condition, given as a discrete number and unit of measurement
☐	☒	A statement on whether measurements were taken from distinct samples or whether the same sample was measured repeatedly
☒	☐	The statistical test(s) used AND whether they are one- or two-sided<br>*Only common tests should be described solely by name; describe more complex techniques in the Methods section.*
☒	☐	A description of all covariates tested
☒	☐	A description of any assumptions or corrections, such as tests of normality and adjustment for multiple comparisons
☒	☐	A full description of the statistical parameters including central tendency (e.g. means) or other basic estimates (e.g. regression coefficient) AND variation (e.g. standard deviation) or associated estimates of uncertainty (e.g. confidence intervals)
☒	☐	For null hypothesis testing, the test statistic (e.g. *F*, *t*, *r*) with confidence intervals, effect sizes, degrees of freedom and *P* value noted<br>*Give P values as exact values whenever suitable.*
☒	☐	For Bayesian analysis, information on the choice of priors and Markov chain Monte Carlo settings
☒	☐	For hierarchical and complex designs, identification of the appropriate level for tests and full reporting of outcomes
☒	☐	Estimates of effect sizes (e.g. Cohen's *d*, Pearson's *r*), indicating how they were calculated

Our web collection on statistics for biologists contains articles on many of the points above.

Software and code

Policy information about availability of computer code

Data collection	EPU 2.14
Data analysis	RELION (v4.0), CTFFIND (v4.1), COOT (v0.9.8.91), ISOLDE (v1.5), REFMAC (v5.8.0387), Servalcat (v0.2.85), ChimeraX (v1.5), PyMol (v2.5.5), GraphPad Prism (v10), MolProbity 4.5.2., ImageJ (v2.14.0)

For manuscripts utilizing custom algorithms or software that are central to the research but not yet described in published literature, software must be made available to editors and reviewers. We strongly encourage code deposition in a community repository (e.g. GitHub). See the Nature Portfolio guidelines for submitting code & software for further information.

Data

Policy information about availability of data

All manuscripts must include a data availability statement. This statement should provide the following information, where applicable:

- Accession codes, unique identifiers, or web links for publicly available datasets
- A description of any restrictions on data availability
- For clinical datasets or third party data, please ensure that the statement adheres to our policy

Cryo-EM maps have been deposited in the Electron Microscopy Data Bank (EMDB) with the accession numbers EMD-51319 for P301L tau filaments from case 2, EMD-51320 for type I P301T tau filaments and EMD-51325 for type II P301T tau filaments. The corresponding refined atomic models have been deposited in the

Protein Data Bank (PDB) under accession numbers 9GG0 for P301L tau filaments from case 2, 9GG1 for type I P301T tau filaments and 9GG5 for type II P301T tau filaments.

Research involving human participants, their data, or biological material

Policy information about studies with human participants or human data. See also policy information about sex, gender (identity/presentation), and sexual orientation and race, ethnicity and racism.

Reporting on sex and gender	See Method section. Two females belonging to two separate US families with mutation P301L in MAPT. Three males belonging to one Dutch family (family 1) with mutation P301L in MAPT. One male from a Spanish family with mutation P301T in MAPT.
Reporting on race, ethnicity, or other socially relevant groupings	Not relevant to study.
Population characteristics	See Method section. Ages at death (years) for P301L cases 1-5: 62, 55, 55, 57, 64, respectively. Age at death for P301T case: 49 years. Two individuals from US, three individuals from the Netherlands, one individual from Spain.
Recruitment	Samples were selected based on neuropathological examination and brain tissue availability, which is unlikely to have impacted the results.
Ethics oversight	The studies carried out at Indiana University, Rotterdam University and University of Barcelona were approved through the ethical review processes at each university's Institutional Review Board (IRB). Informed consent was obtained from the patients' next of kin

Note that full information on the approval of the study protocol must also be provided in the manuscript.

Field-specific reporting

Please select the one below that is the best fit for your research. If you are not sure, read the appropriate sections before making your selection.

☒ Life sciences ☐ Behavioural & social sciences ☐ Ecological, evolutionary & environmental sciences

For a reference copy of the document with all sections, see nature.com/documents/nr-reporting-summary-flat.pdf

Life sciences study design

All studies must disclose on these points even when the disclosure is negative.

Sample size	We used parietal cortex from two cases belonging to two separate US families with mutation P301L in MAPT. We also used temporal cortex from three Dutch cases belonging to one extended family with mutation P301L in MAPT. Furthermore, we used frontal cortex from a previously described case from a Spanish family with mutation P301T in MAPT. Samples were chosen based on availability and neuropathological examination.
Data exclusions	Pre-established common image classification procedures (Scheres 2012. J. Struc. Biol. 180, 519-530) were employed to select particle images with the highest resolution content in the cryo-EM reconstruction process. Details of the number of selected images are given in Extended Data Table 1.
Replication	For P301L, all attempts at replication were successful. Four independent biological repeats per experiment where representative data are shown, as described in the main text. For P301T, we analysed only one case due to availability.
Randomization	Because there is no assignment of data points to distinct groups, randomisation was not applicable to this study.
Blinding	The investigators were not blinded to allocation during experiments and outcome assessment. The perceived risk of detection/performance bias was deemed negligible.

Reporting for specific materials, systems and methods

We require information from authors about some types of materials, experimental systems and methods used in many studies. Here, indicate whether each material, system or method listed is relevant to your study. If you are not sure if a list item applies to your research, read the appropriate section before selecting a response.

Materials & experimental systems

n/a	Involved in the study
☐	☒ Antibodies
☒	☐ Eukaryotic cell lines
☒	☐ Palaeontology and archaeology
☒	☐ Animals and other organisms
☒	☐ Clinical data
☒	☐ Dual use research of concern
☒	☐ Plants

Methods

n/a	Involved in the study
☒	☐ ChIP-seq
☒	☐ Flow cytometry
☒	☐ MRI-based neuroimaging

Antibodies

Antibodies used

Primary antibodies used are presented in the Methods section and in Extended Data Table 1.
BR133 (in-house, diluted 1:4'000 for WB);
RD3 (Millipore, #05-803, diluted 1:4'000 for WB and 1:3'000 for histology);
Anti-4R (Cosmo Bio, #CAC-TIP-4RT-P01, diluted 1:2'000 for WB and 1:400 for histology);
BR134 (in-house, diluted 1:4'000 for WB);
AT8 (Thermo Fisher, #MN1020, diluted 1:11'000 for WB and 1:1'000 for histology);
AT100 (Thermo Fisher, #MN1060, diluted 1:500 for WB).

Validation

BR134 validated against the C-terminus of human tau in [Goedert et al. 1989 Neuron 3,519-526];
RD3 validated against human 3R tau in manufacturer's datasheet (Millipore);
Anti-4R validated against human tau residues 275-291 in [Falcon et al. 2018 Nature 561,137-140] and validated for Western blot and IHC(P), this isoform-specific tau antibody is useful for immunohistochemical and biochemical studies of tau species in diverse neurodegenerative diseases (Cosmo Bio);
BR134 validated against the N-terminus of human tau in [Goedert et al. 1989 Neuron 3,519-526];
AT8 validated against human ptau pS202 and pT205 in manufacturer's datasheet (Thermo Fisher). This antibody was validated by cell treatment to ensure the antibody binds to the antigen stated;
AT100 validated against human tau pT212 and pS214 in manufacturer's datasheet (Thermo Fisher).

Plants

Seed stocks

Not relevant to study.

Novel plant genotypes

Not relevant to study.

Authentication

Not relevant to study.
