# Supplementary figures and images for: Distinct tau filament folds in human MAPT mutants P301L and P301T
Source: Nat Struct Mol Biol. 2025 May 29;32(8):1470–8. doi: 10.1038/s41594-025-01575-9 (PMC12350173; doi:10.1038/s41594-025-01575-9)

Source Data Figure 1

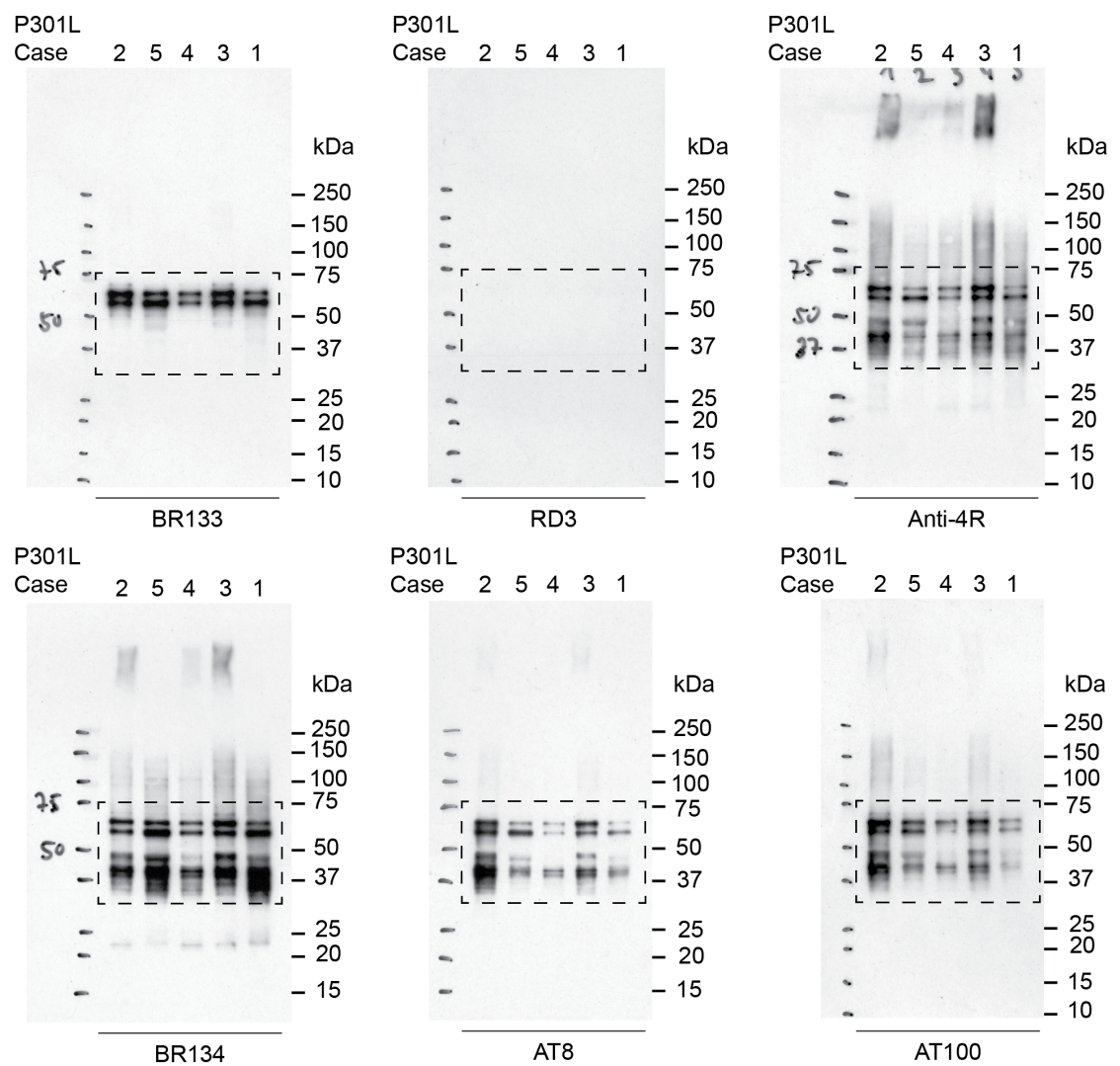

Source images for Western blots shown in Figure 1b.

Supplement: Supplementary file 3 — Uncropped western blots for Fig. 1b. [file 41594_2025_1575_MOESM3_ESM.pdf]

Source Data Figure 3b

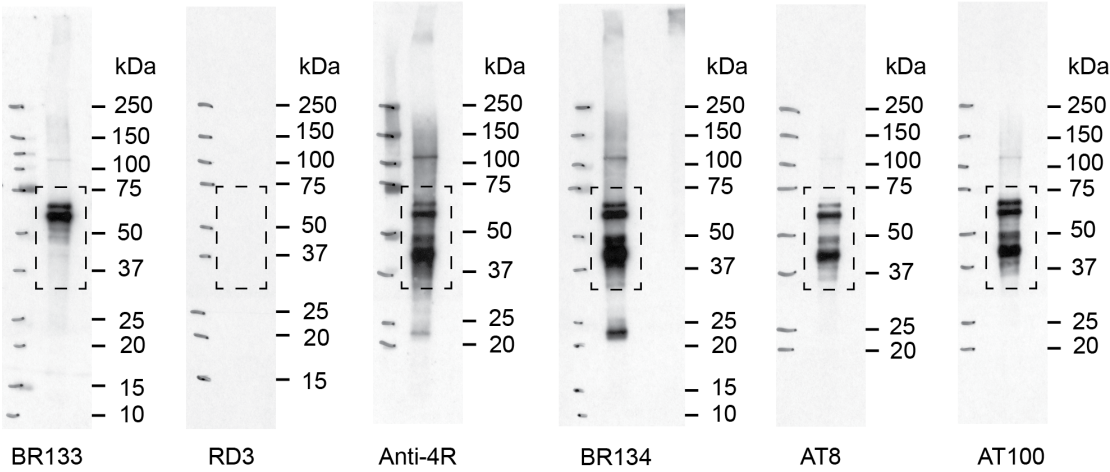

Supplement: Supplementary file 4 — Uncropped western blots for Fig. 3b. [file 41594_2025_1575_MOESM4_ESM.pdf]
